# Genomic Surveillance of Endemic Human Coronaviruses in Côte d’Ivoire Using Targeted Hybrid-Capture Sequencing

**DOI:** 10.3390/v18060678

**Published:** 2026-06-17

**Authors:** Ange-Michèle M’bra, Syndou Meite, Herve A. Kadjo, Luc Venance Kouakou, Yakoura Ouattara, Mouhamed Kane, Helene A. Kouassi, Ndeye Awa Ndiaye, Olivia Cariolh Koumba-Koumba, Alida Mouliom, Safiétou Sankhe, David Coulibaly Ngolo, Ndongo Dia, Edgard Adjogoua, Moussa Moise Diagne

**Affiliations:** 1 Department of Epidemic Viruses, Pasteur Institute of Côte d’Ivoire, Abidjan 01 BP 490, Côte d’Ivoire; angemichelembra@gmail.com (A.-M.M.); rvkdjo@yahoo.fr (H.A.K.); o.kady@yahoo.fr (Y.O.); agbessitherese@gmail.com (H.A.K.); cariolhkoumba@gmail.com (O.C.K.-K.); alidamouliom@gmail.com (A.M.); edgarvalery@yahoo.fr (E.A.); 2 Molecular Biology Platform, Pasteur Institute of Côte d’Ivoire, Abidjan 01 BP 490, Côte d’Ivoire; syndoumeite@pasteur.ci (S.M.); davidcoulibalyngolo@gmail.com (D.C.N.); 3 Department of Biochemistry and Genetics, Peleforo Gon Coulibaly University, Korhogo BP 1328, Côte d’Ivoire; lucvenance0@yahoo.fr; 4 Virology Department, Institut Pasteur de Dakar, 36 Avenue Pasteur, Dakar BP 220, Senegal; mohamed.kane-ext@pasteur.sn (M.K.); ndeyeawa.ndiaye@pasteur.sn (N.A.N.); safietou.sankhe@pasteur.sn (S.S.); ndongo.dia@pasteur.sn (N.D.)

**Keywords:** endemic human coronaviruses, genomic surveillance, Côte d’Ivoire, hybrid-capture sequencing, respiratory viruses, phylogenetic analysis

## Abstract

Endemic human coronaviruses (HCoVs) are important contributors to respiratory infections, yet genomic data from sub-Saharan Africa remain limited. We analyzed 13,530 nasopharyngeal samples collected through the national influenza sentinel surveillance network in Côte d’Ivoire between 2022 and 2024 to characterize the circulation and genomic diversity of endemic HCoVs. A subset of 52 RT-qPCR-positive samples with Ct values ≤ 28 was selected for targeted hybrid-capture sequencing using the Twist Bioscience Respiratory Virus Research Panel. Genome recovery metrics were available for 28 samples, including HCoV-NL63 (*n* = 9), HCoV-229E (*n* = 8), HCoV-OC43 (*n* = 9), and HCoV-HKU1 (*n* = 2). Endemic HCoVs circulated throughout the study period, with temporal variation across species and increased detections during several rainy-season months. No co-presence of multiple endemic HCoV species was identified in the final analytical dataset. Genome recovery differed by species, with broader and more consistent coverage for HCoV-OC43 and HCoV-NL63 than for HCoV-229E and HCoV-HKU1. Phylogenetic analysis showed that all recovered HCoV-229E genomes clustered within genotype L6 and all recovered HCoV-HKU1 genomes within genotype A, whereas HCoV-OC43 and HCoV-NL63 were distributed across multiple genotypes among recovered genomes. To our knowledge, these findings provide the first genomic data on endemic HCoVs from Côte d’Ivoire and support the feasibility and further targeted integration of targeted hybrid-capture sequencing into routine genomic surveillance of respiratory viruses.

## 1. Introduction

Endemic human coronaviruses (HCoVs) belong to the genera Alphacoronavirus (HCoV-229E, HCoV-NL63) and Betacoronavirus (HCoV-OC43, HCoV-HKU1). Long before the emergence of SARS-CoV-2, these viruses circulated seasonally worldwide and were recognized as causes of acute respiratory infections ranging from mild upper respiratory illness to more severe disease in infants, older adults, and immunocompromised individuals [1,2,3,4,5]. Their evolutionary, antigenic, and replication features have been described across several HCoV species, including HCoV-229E and HCoV-OC43 [6,7,8,9,10,11]. Respiratory virome and respiratory virus surveillance studies further emphasize the importance of monitoring common respiratory viruses across age groups and surveillance contexts, including in the post-pandemic period [12,13,14,15]. However, genomic surveillance of endemic HCoVs remains limited in many African settings, including Côte d’Ivoire. Although next-generation sequencing has transformed respiratory virus surveillance and has become an important component of genomic surveillance strategies [16,17], genome recovery can be challenging in routine clinical samples with low viral loads or degraded RNA, leading to incomplete assemblies and reduced phylogenetic resolution [18,19,20,21,22]. Targeted hybrid-capture enrichment and related targeted sequencing approaches have emerged as complementary strategies for improving viral genome recovery, including from low-titer samples, by enriching target viral nucleic acids prior to sequencing [23,24,25,26,27,28,29,30]. In Côte d’Ivoire, strengthening genomic surveillance of respiratory viruses has become an important public health objective, particularly since the COVID-19 pandemic [17]. In Africa, endemic HCoVs contribute to influenza-like illness and severe acute respiratory infection, with variable positivity rates across age groups, clinical syndromes, diagnostic panels, and surveillance designs [31,32]. However, most regional data remain based on molecular detection rather than whole-genome characterization, and publicly available endemic HCoV genomes from West Africa remain sparse. This limits the ability to compare genotype circulation, lineage turnover, and genome recovery performance across African settings. The objectives of this study were to: (i) describe the detection patterns of HCoV-229E, HCoV-NL63, HCoV-OC43, and HCoV-HKU1 in sentinel respiratory samples collected in Côte d’Ivoire between 2022 and 2024; (ii) evaluate genome recovery by species and Ct value using targeted hybrid-capture sequencing; and (iii) determine the genotype placement of recovered endemic HCoV genomes in relation to representative global reference sequences.

## 2. Materials and Methods

### 2.1. Study Setting

This study was conducted within the national sentinel surveillance system for influenza and other respiratory viruses in Côte d’Ivoire. The system, coordinated by national public health authorities, includes ten sentinel healthcare facilities distributed across the country to improve the geographic representativeness of epidemiological data. Five sites are located in Abidjan: Angré University Hospital, Treichville University Hospital, Attécoubé General Hospital, Yopougon General Hospital, and the Abidjan Penitentiary Health Facility. The remaining five sites are located in Bouaké, Korhogo, Man, San Pedro, and Agnibilékro. This network supports systematic collection of respiratory specimens from patients meeting the case definition for acute respiratory infection in order to monitor respiratory virus circulation, support early detection of emerging pathogens, and contribute to the molecular characterization of circulating strains. Patients were enrolled through the system if they met the surveillance case definition for influenza-like illness (ILI) or severe acute respiratory infection (SARI). ILI was defined as acute respiratory illness with measured fever of ≥38 °C and cough, with symptom onset within the previous 10 days, whereas SARI was defined as ILI requiring hospitalization. Outpatient cases were sampled through sentinel healthcare facilities, while hospitalized cases were included through SARI surveillance pathways. Specimen collection was systematic among eligible patients meeting ILI/SARI criteria.

### 2.2. Statistical Analysis

Sociodemographic and surveillance data collected over the 2022–2024 study period were summarized using frequencies, proportions, and positivity rates. Age-specific positivity was calculated by dividing the number of HCoV-positive samples by the total number tested in each age group, and 95% confidence intervals (95% CIs) were estimated using binomial methods. Annual positivity was calculated using the number of HCoV-positive samples divided by the total number of respiratory samples tested each year. Because individual-level covariates and complete denominator data by month, site, sex, and clinical syndrome were not available for all analyses, epidemiological comparisons were interpreted descriptively. Accordingly, we did not emphasize age-only odds ratios as evidence of differential risk. Monthly HCoV detections were plotted to describe temporal patterns; however, monthly counts were not interpreted as monthly positivity rates unless the corresponding monthly number of tested samples was available. All statistical analyses and graphical visualizations were performed using R software (version 4.4.3) and Microsoft Excel.

### 2.3. Sample Collection and Molecular Detection

Nasopharyngeal swabs were collected between January 2022 and December 2024 through the sentinel surveillance network. Specimens were stored in universal transport medium (UTM^®^, Copan Diagnostics, Corona, CA, USA) and transported to the National Reference Laboratory for analysis. Viral RNA extraction was performed either using the automated KingFisher™ Flex system (Thermo Fisher Scientific, Waltham, MA, USA) or manually with the QIAamp Viral RNA Mini Kit (QIAGEN, Hilden, Germany), according to the manufacturer’s instructions. Detection of the four endemic human coronaviruses (HCoV-229E, HCoV-NL63, HCoV-OC43, and HCoV-HKU1) was carried out by real-time reverse transcription polymerase chain reaction (RT-qPCR) using previously published protocols: HCoV-OC43 [33], HCoV-229E and HCoV-NL63 [34], and HCoV-HKU1 [4]. Assays were implemented locally on Bio-Rad CFX96 and QuantStudio 5 real-time PCR platforms using the SuperScript III Platinum One-Step qRT-PCR kit (Invitrogen, Carlsbad, CA, USA), following the published protocols cited above. Each sample was tested for the four endemic HCoVs within the same diagnostic workflow. A uniform positivity threshold of Ct ≤ 37 was applied across the four HCoV targets. Samples with no amplification or Ct values above this threshold in a valid run were considered negative. Indeterminate results were systematically repeated to assign a final positive or negative status. Each amplification run included an in-house positive control prepared from previously confirmed HCoV-positive samples, a no-template control consisting of RNase-free water, and an internal control to validate RNA extraction efficiency and exclude PCR inhibition.

### 2.4. Sample Selection for Genomic Sequencing

Targeted sampling was performed among confirmed HCoV-positive cases to optimize the success of whole-genome sequencing. A total of 52 samples were selected based on a cycle-threshold (Ct) value ≤ 28, a criterion chosen to maximize the likelihood of adequate sequencing depth and genome coverage. This Ct threshold was applied to the species-specific positive RT-qPCR target for each HCoV species using the same selection principle across HCoV-229E, HCoV-NL63, HCoV-OC43, and HCoV-HKU1. Samples were not intentionally balanced by species or by year; selection prioritized viral load, sample availability, and inclusion of all four endemic HCoV species where possible. Samples with lower viral loads are more frequently associated with incomplete coverage, missing genomic regions, and genome assembly failure. This strategy optimized available analytical resources and increased the probability of generating sequences suitable for genomic characterization. However, because the sequenced subset was restricted to high-viral-load specimens, it should not be interpreted as fully representative of all HCoV-positive samples detected during the study period.

### 2.5. Hybrid-Capture Sequencing

Extracted viral nucleic acids were converted into double-stranded DNA (dsDNA) through first- and second-strand synthesis using the ProtoScript II Reaction Mix and Enzyme Mix and the NEBNext Second Strand Synthesis Kit, according to the manufacturers’ instructions. The resulting dsDNA was purified with AMPure XP beads and quantified using a Qubit™ fluorometer (Thermo Fisher Scientific, Waltham, MA, USA).

Sequencing libraries were prepared from 50 ng of purified dsDNA per sample using the Twist Library Preparation EF 2.0 workflow with enzymatic fragmentation and the Twist Universal Adapter System (Twist Bioscience, San Francisco, CA, USA), including fragmentation for 20 min at 37 °C, end repair, 3′ adenylation, adapter ligation, and library amplification. Indexed libraries were amplified using 6 pre-capture PCR cycles. Library quality and concentration were assessed prior to target enrichment. Target enrichment was performed by solution-based hybrid capture using the Twist Respiratory Virus Research (RVR) Panel (103067, Twist Bioscience). Indexed libraries were pooled (8 samples per pool; up to 1.5 µg DNA per pool), hybridized with capture probes at 70 °C for 16 h, recovered with streptavidin-coated magnetic beads, and amplified post-capture using 15 PCR cycles to enrich viral sequences. Enriched libraries were purified, quantified, and validated using the Qubit HS Assay before sequencing on the Illumina iSeq 100 platform (Illumina, San Diego, CA, USA) using the iSeq 100 i1 Reagent v2 300-cycle kit in paired-end mode (2 × 150 bp). The resulting data were used for downstream genomic characterization and phylogenetic analysis of circulating HCoV strains.

### 2.6. Bioinformatic and Phylogenetic Analysis

Raw sequencing files in FASTQ format generated on the Illumina iSeq 100 platform were uploaded to the CZ ID (Chan Zuckerberg ID) [35], a cloud-based platform for metagenomic sequencing analysis. Samples were processed using the integrated short-read metagenomic pipeline, with workflow version 8, including quality control, adapter trimming, and host-read subtraction using default parameters. The CZ ID Illumina workflow performs read-level quality control, adapter trimming, removal of low-quality and low-complexity reads, host/human read subtraction, duplicate-read handling, and taxonomic classification of non-host reads. Quality filtering is based on a customized fastp workflow that removes bases with quality scores below 17, reads shorter than 35 bp, reads with more than 15 undetermined bases, and reads with high low-complexity content. Host and human read removal are performed using Bowtie2 and HISAT2-based filtering, and duplicate reads are identified using CZID-dedup.

For the targeted analyses presented here, CZ ID species-level outputs were reviewed for each sample, and sample-level metrics were extracted, including total reads, HCoV-mapped reads, mapped-read proportion, genome breadth of coverage, and mean sequencing depth. Mapped-read proportion was calculated as HCoV-mapped reads divided by total reads × 100.

Consensus FASTA sequences were generated from the species-specific CZ ID viral consensus outputs using the corresponding HCoV reference accession selected in CZ ID. In this workflow, non-human reads are aligned to the selected viral reference using minimap2, aligned reads are trimmed using Trim Galore, and consensus sequences are called using iVar consensus. Positions not meeting base-calling criteria are reported as N, and ambiguous sites are represented using IUPAC ambiguity codes when more than one nucleotide is supported. A standard base is called when a single nucleotide reaches >75% frequency; otherwise, the position is treated as ambiguous.

Genome breadth of coverage was defined as the percentage of the reference genome recovered in the consensus sequence. Mean depth was defined as the average read depth across the corresponding HCoV reference genome. Genome recovery was categorized as high when genome breadth was ≥80% with mean depth ≥ 10×, intermediate when genome breadth was 20–79% or when genome breadth was ≥80% but mean depth was <10×, and low when genome breadth was <20%. These thresholds were selected to distinguish genomes suitable for genome-scale interpretation from partial or low-confidence recoveries. Sequencing-performance analyses included all samples with available genome recovery metrics, whereas phylogenetic and genotype interpretation was restricted to genomes with sufficient recovery for reliable placement.

FASTA sequences generated were supplemented with representative reference sequences for each seasonal HCoV species retrieved from publicly available databases, including NCBI GenBank, to provide phylogenetic context. Reference sequences were selected to represent recognized genotype diversity, geographic and temporal diversity, and publicly available genomes clustering near the Côte d’Ivoire sequences. The full list of reference sequences used for phylogenetic reconstruction, including accession number, virus species, country/location, year, genotype, sequence length, and rationale for inclusion, is provided in Supplementary Table S1.

Multiple sequence alignment was performed using MAFFT [36], and alignment quality was visually inspected, with ambiguously aligned regions removed where necessary prior to phylogenetic inference.

Phylogenetic reconstruction was performed using the Maximum Likelihood (ML) method [37] using IQ-Tree [38]. The nucleotide substitution model was selected using ModelFinder [39], and branch support was evaluated by bootstrap resampling with 1000 standard non-parametric bootstrap replicates.

Preliminary tree topologies were examined in FigTree [40], and genotype assignment was based on clustering with representative reference sequences. Final phylogenetic tree visualization and annotation were conducted in R v4.6.0 using the ggtree v4.3.0 and ggplot2 v4.0.3 packages.

## 3. Results

### 3.1. Patterns of HCoV Detection and Circulation

During the study period (2022–2024), 13,530 respiratory samples were analyzed for human coronaviruses (HCoVs), of which 439 were positive, corresponding to an overall positivity rate of 3.24%. Annual testing denominators were 3998 samples in 2022, 4323 in 2023, and 5209 in 2024. The corresponding annual HCoV positivity rates were 2.38% in 2022, 3.54% in 2023, and 3.67% in 2024. Thus, the increase in HCoV detections over the study period was not explained solely by increased testing volume, although interpretation remains limited by the absence of fully stratified monthly and site-specific denominators. Children aged 1–5 years represented the largest proportion of tested individuals (34.1%) and of HCoV-positive cases (35.3%), followed by infants aged <1 year (22.6%) and young adults aged 16–35 years (18.2%). The highest positivity rate was observed among individuals aged 16–35 years (3.57%), followed by children aged 1–5 years (3.35%) and infants aged <1 year (3.30%). Lower positivity rates were observed among school-aged children aged 6–15 years (2.37%) and adults aged >65 years (2.55%). Age-specific positivity varied modestly across groups, ranging from 2.37% among children aged 6–15 years to 3.57% among individuals aged 16–35 years. However, these differences were interpreted descriptively, and no age group was considered to have a statistically supported higher risk of HCoV detection based on the available data. Because the >65-year age group included only 13 positive cases, we avoided overemphasis on age-only odds ratios and instead focused on unadjusted positivity estimates with 95% confidence intervals (Table 1).

**Figure 1:**
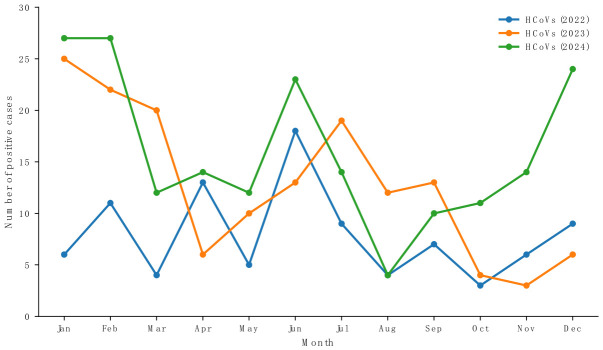
Monthly detection counts of endemic human coronaviruses identified through sentinel respiratory surveillance in Côte d’Ivoire, 2022–2024. Values represent monthly numbers of HCoV-positive samples.

Monthly analysis of HCoV detections revealed marked temporal variability over the three-year period (Figure 1). In 2022, detections remained relatively low to moderate, ranging from 3 to 18 cases per month, with a first increase in February (*n* = 11), another in April (*n* = 13), and a peak in June (*n* = 18), followed by a decline from August to October (*n* = 4, 7, and 3, respectively). In 2023, HCoV activity was highest at the beginning of the year, with 25 cases in January, 22 in February, and 20 in March, then declined to 6 cases in April before increasing again in June (*n* = 13) and July (*n* = 19); detections subsequently decreased to their lowest levels in October (*n* = 4) and November (*n* = 3), before rising slightly in December (*n* = 6). In 2024, circulation was elevated early in the year, with 27 cases in both January and February, decreased in March–May (*n* = 12, 14, and 12, respectively), rose again in June (*n* = 23), and remained detectable through the second half of the year, with a marked increase in December (*n* = 24). HCoV detections occurred throughout the year, with temporal heterogeneity across months and years. Although increases were observed during some months that may overlap with rainy-season periods, these patterns should be interpreted cautiously because monthly and site-specific testing denominators were not available, rainy-season periods were not formally defined for each study region, and no denominator-adjusted seasonal model was performed. Therefore, the present data describe temporal variation in detection counts rather than confirmed seasonal dynamics.

**Figure 2:**
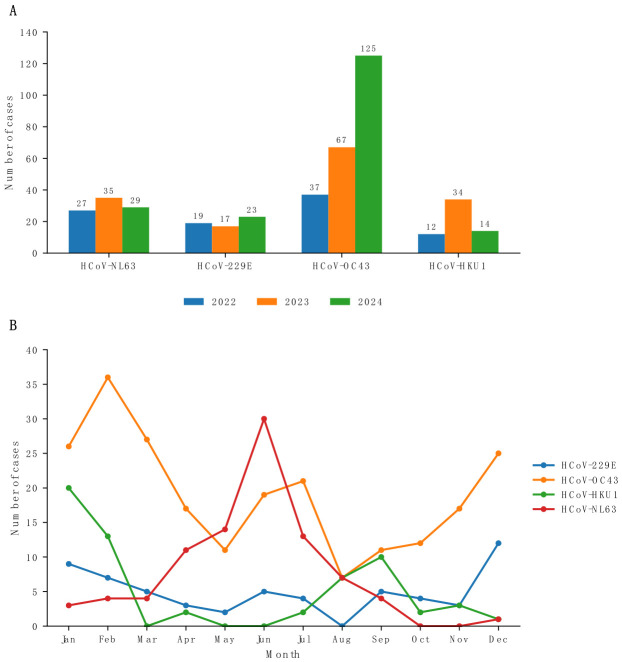
Annual and aggregated monthly distribution of endemic human coronavirus species detected from 2022 to 2024. (**A**) Yearly number of cases caused by HCoV-229E, HCoV-NL63, HCoV-OC43, and HCoV-HKU1. (**B**) Aggregated monthly distribution of these four HCoV species over the study period.

To further characterize species-specific viral dynamics, we examined the annual and aggregated monthly distribution of endemic HCoVs (Figure 2A,B). Among the 439 HCoV-positive samples, betacoronaviruses predominated, accounting for 289/439 cases (65.8%), whereas alphacoronaviruses accounted for 150/439 cases (34.2%). As shown in Figure 2A, HCoV-OC43 was the most frequently detected species and increased markedly over time, from 37 cases in 2022 to 67 in 2023 and 125 in 2024. HCoV-NL63 showed moderate interannual variability, with 27, 35, and 29 cases in 2022, 2023, and 2024, respectively, while HCoV-229E remained relatively stable across the study period, with 19, 17, and 23 cases, respectively. HCoV-HKU1 increased from 12 cases in 2022 to 34 in 2023, before declining to 14 in 2024 (Figure 2A). When monthly detections were aggregated across years, Figure 2B showed that HCoV-OC43 circulated throughout the year, HCoV-NL63 displayed a pronounced peak in June, HCoV-229E remained at comparatively low levels with limited monthly fluctuation, and HCoV-HKU1 was detected predominantly during the early months of the year. Together, these findings highlight the predominance of HCoV-OC43 and distinct temporal circulation patterns among endemic HCoV species. A sample-level review of the species-specific RT-qPCR results did not identify co-presence of multiple endemic HCoV species in any specimen included in the final analytical dataset. Accordingly, the species-specific counts reported here were treated as mutually exclusive detections, and the sum of HCoV-229E, HCoV-NL63, HCoV-OC43, and HCoV-HKU1 detections corresponded to the total number of HCoV-positive samples analyzed. This clarification addresses the distinction between the co-circulation of several HCoV species at the population level and the co-detection of multiple HCoV species within the same individual sample.

However, HCoV-OC43 positivity also increased when expressed relative to annual testing denominators, from 0.93% in 2022 to 1.55% in 2023 and 2.40% in 2024. This suggests that the observed rise in HCoV-OC43 detections may not be attributable only to increased sample numbers. Nevertheless, month- and site-specific denominators would be required to determine whether this reflected a true change in circulation intensity, changes in sampling patterns, or both.

### 3.2. Sequencing Performance Across HCoV Species

A total of 52 RT-qPCR-positive samples with Ct values ≤ 28 were selected for targeted hybrid-capture sequencing. The 52 selected samples included HCoV-NL63 (*n* = 14), HCoV-229E (*n* = 10), HCoV-OC43 (*n* = 18), and HCoV-HKU1 (*n* = 10). By year, 24 samples were selected from 2022, 11 from 2023, and 17 from 2024. The species-by-year distribution is shown in Table 2.

**Figure 3:**
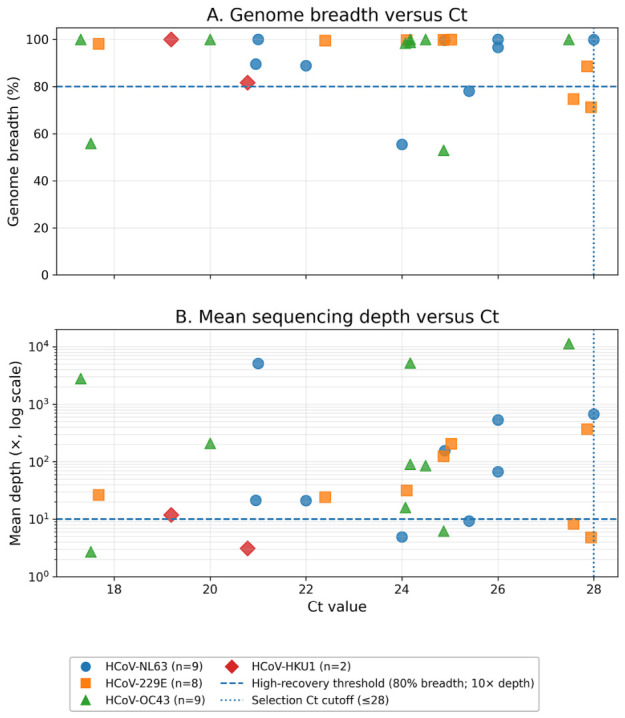
Sequencing performance by Ct value across endemic HCoV species. (**A**) Genome breadth of coverage (%) according to Ct value. The dashed horizontal line indicates the 80% breadth threshold used for the high recovery classification, and the vertical dotted line indicates the Ct selection threshold of ≤28. (**B**) Mean sequencing depth according to Ct value, displayed on a log scale. The dashed horizontal line indicates the 10× mean-depth threshold used for high recovery classification. Each point represents one sequenced sample with available genome recovery metrics.

Sequencing performance varied across HCoV species (Figure 3, Supplementary Table S2). Run-level quality metrics were: projected total yield, 1.7 Gb; Q30 Read 1, 90.5%; Q30 Read 2, 80.5%; clusters passing filter, 71.3%; and occupancy, 90.1%. Among the 52 RT-qPCR-positive samples selected for targeted hybrid-capture sequencing, genome recovery metrics suitable for sequencing-performance analysis were available for 28 samples, including HCoV-NL63 (*n* = 9), HCoV-229E (*n* = 8), HCoV-OC43 (*n* = 9), and HCoV-HKU1 (*n* = 2). Across these 28 samples, the median number of total reads was 21,626, the median number of HCoV-mapped reads was 9999, and the median mapped-read proportion was 76.8%. Using the predefined recovery criteria, 21/28 samples were classified as high recovery and 7/28 as intermediate recovery, with no low recovery (Supplementary Table S2).

By species, high/intermediate/low recovery counts were 7/2/0 for HCoV-NL63, 6/2/0 for HCoV-229E, 7/2/0 for HCoV-OC43, and 1/1/0 for HCoV-HKU1. Median Ct, genome breadth, and mean depth were 24.9 (IQR: 22.0–26.0), 96.7% (IQR: 88.9–99.9), and 67.4 × (IQR: 21.0–533.3) for HCoV-NL63; 25.0 (IQR: 23.7–27.6), 98.8% (IQR: 85.1–99.7), and 29.0× (IQR: 20.2–144.4) for HCoV-229E; and 24.2 (IQR: 20.0–24.5), 100.0% (IQR: 98.5–100.0), and 89.9× (IQR: 16.0–2778.8) for HCoV-OC43. For HCoV-HKU1, only two samples had genome recovery metrics; median Ct, genome breadth, and mean depth were 20.0, 90.9%, and 7.5×, respectively. Across the 28 samples, Ct value showed no statistically significant correlation with genome breadth (Spearman’s ρ = −0.10, *p* = 0.61) or mean depth (Spearman’s ρ = 0.20, *p* = 0.31), suggesting that sequencing performance was not explained by Ct value alone and may also reflect species-specific or sample-level factors.

**Figure 4:**
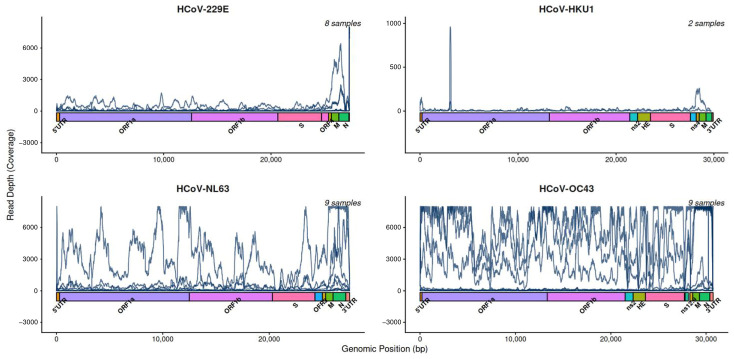
Comparative read-depth profiles across the genomes of four human coronaviruses. Panels show sequencing coverage across the genomes of HCoV-229E, HCoV-HKU1, HCoV-NL63, and HCoV-OC43. The x-axis indicates genomic coordinates and the y-axis shows read depth. Colored bars below each panel represent the genomic organization, including ORF1ab, S, M, and N. The number of analyzed samples is indicated in the upper-right corner of each panel.

Read-depth profiles across the genome also revealed marked inter-species differences (Figure 4). HCoV-OC43 and HCoV-NL63 showed higher and more even coverage across large portions of the genome, whereas HCoV-229E and HCoV-HKU1 had lower depth and less homogeneous coverage. Coverage also appeared to increase toward the 3′ end of the genome in several profiles, consistent with known biases in coronavirus RNA sequencing. These profiles helped assess the suitability of recovered genomes for consensus generation and downstream phylogenetic analysis.

### 3.3. Phylogenetic Analysis

**Figure 5:**
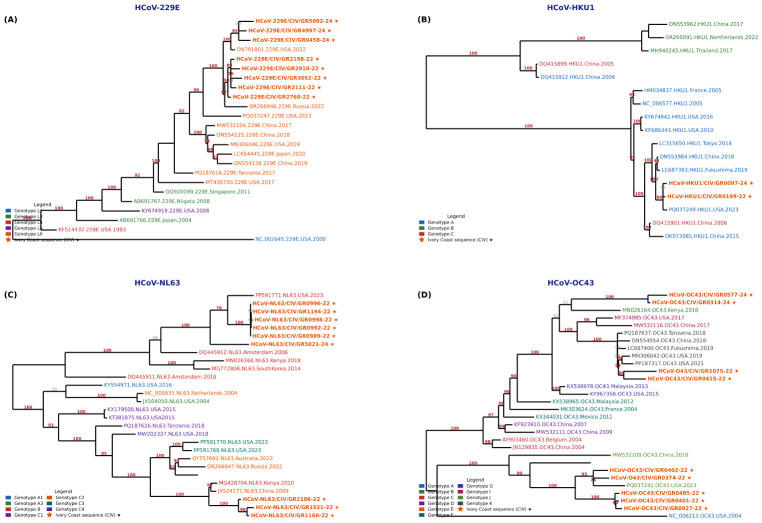
Phylogenetic placement of endemic human coronavirus sequences detected in Côte d’Ivoire. Maximum-likelihood phylogenetic trees were generated for four endemic human coronaviruses: HCoV-229E (**A**), HCoV-HKU1 (**B**), HCoV-NL63 (**C**), and HCoV-OC43 (**D**). Côte d’Ivoire sequences are highlighted in orange and marked with an asterisk, while reference sequences are labeled by accession number, virus species, country, and year when available. Genotypes are indicated by different colors according to the legend in each panel. Bootstrap support values are shown at the main nodes, and branch lengths are proportional to the number of nucleotide substitutions per site. The analysis shows that Côte d’Ivoire sequences clustered within previously described global genotype diversity, indicating the circulation of multiple endemic HCoV lineages during the study period.

Phylogenetic analysis revealed marked species-specific differences in the placement of recovered endemic HCoV genomes circulating in Côte d’Ivoire during 2022–2024 (Figure 5). All recovered HCoV-229E genomes clustered within genotype L6 (Figure 5A), and all recovered HCoV-HKU1 genomes grouped within genotype A (Figure 5B), indicating relatively limited lineage diversity for these two species in the sampled population. In contrast, HCoV-NL63 genomes were distributed across genotypes B and C2 (Figure 5C), while HCoV-OC43 genomes clustered within genotypes F, I, and J (Figure 5D), consistent with the co-circulation of multiple genetically distinct lineages. Collectively, these findings demonstrate heterogeneous evolutionary patterns among endemic HCoVs, with HCoV-OC43 and HCoV-NL63 exhibiting broader phylogenetic diversity than HCoV-229E and HCoV-HKU1 during the study period.

Taken together, the temporal, sequencing, and phylogenetic findings point to important species-specific differences in the epidemiology and evolutionary dynamics of endemic HCoVs circulating in Côte d’Ivoire. These genotype patterns should be interpreted as reference-supported phylogenetic placements among recovered genomes, particularly for HCoV-HKU1, rather than as definitive evidence of population-wide lineage diversity.

## 4. Discussion

This study provides new genomic insight into the circulation of endemic human coronaviruses in Côte d’Ivoire between 2022 and 2024, based on sentinel respiratory surveillance and whole-genome or near-complete genome sequencing. Rather than restating the temporal, species-specific, and sequencing results in detail, the main contribution of this work is to show that endemic HCoVs circulated in this West African surveillance setting, that HCoV-OC43 was the predominant detected species, and that targeted hybrid-capture sequencing enabled recovery of genomes suitable for phylogenetic placement from selected high-viral-load respiratory samples. Together, these results establish an initial genomic baseline for endemic HCoVs in Côte d’Ivoire and highlight the value of integrating genomic surveillance into routine respiratory virus monitoring in West Africa.

Our findings are consistent with previous respiratory-virus surveillance studies from Africa and other tropical settings, including Ghana, Senegal, Malawi, Cameroon, and Burkina Faso, where endemic HCoVs have been detected among patients with influenza-like illness or severe acute respiratory infection [41,42,43,44,45,46]. Across these studies, reported HCoV positivity rates, age distributions, seasonal patterns, and species predominance vary substantially. These differences likely reflect heterogeneity in surveillance design, geographic coverage, age structure, clinical case definitions, sampling intensity, diagnostic panels, and study periods. Therefore, direct comparison of positivity rates should be made cautiously. In the present study, the overall positivity rate and the predominance of HCoV-OC43 support the contribution of endemic HCoVs to respiratory-virus circulation in Côte d’Ivoire, but the data should be interpreted as evidence from the studied sentinel population rather than as a nationally representative estimate of HCoV burden. This interpretation is aligned with WHO guidance for integrated sentinel respiratory-virus surveillance, which emphasizes standardized surveillance processes and denominator-based interpretation of positivity trends [47].

The temporal distribution of detections showed that endemic HCoVs circulated throughout the year, with recurrent increases during several rainy-season months. However, because complete monthly and site-specific testing denominators were not available, these patterns should be interpreted as detection counts rather than formal monthly positivity rates. Although the intensity of circulation varied across years, these patterns suggest that HCoV activity in Côte d’Ivoire may follow seasonal dynamics that differ from the winter-dominant patterns commonly described in temperate regions and may be influenced by local meteorological and respiratory-virus transmission contexts [48,49]. This interpretation should remain cautious, because the present analysis did not include a denominator-adjusted seasonal model or meteorological covariates.

Species-specific analysis further showed that this temporal signal was not uniform across viruses. HCoV-OC43 was the predominant species and increased substantially over the study period, whereas HCoV-NL63, HCoV-229E, and HCoV-HKU1 displayed distinct annual and monthly distributions. When expressed relative to annual testing denominators, HCoV-OC43 positivity increased from 0.93% in 2022 to 1.55% in 2023 and 2.40% in 2024, suggesting that the rise in detections may not be attributable only to increased annual sample numbers. Nevertheless, month- and site-specific denominators would be required to determine whether this reflected a true change in circulation intensity, changes in sampling patterns, or both. The broad circulation of HCoV-OC43, the more pronounced mid-year peak of HCoV-NL63, the relatively low but persistent detection of HCoV-229E, and the more limited distribution of HCoV-HKU1 together point to species-specific circulation patterns rather than a single uniform seasonal profile. These patterns should also be interpreted in the broader context of respiratory virus interactions that may influence observed population dynamics [50].

The absence of multi-HCoV co-detection in the final analytical dataset is notable because simultaneous detection of more than one endemic HCoV has been described, although generally rarely, in other studies using multiplex or parallel RT-qPCR approaches. Studies from Ghana, Senegal, and Malawi documented a small number of specimens positive for two or more seasonal HCoVs, while co-detection of an endemic HCoV with non-coronavirus respiratory viruses was often more frequent [40,41,42]. In our Côte d’Ivoire dataset, the main pattern was therefore co-circulation of multiple HCoV species across the surveillance population rather than frequent co-presence of multiple HCoVs within individual specimens. This distinction is important because co-detection within a sample does not necessarily imply equal contribution of each virus to clinical disease. This interpretation should be distinguished from broader viral co-detection: because the bait-enrichment approach targeted a wide respiratory-virus panel, the analysis also allowed assessment of co-detection between endemic HCoVs and other respiratory viruses.

The sequencing results add an important technical dimension to these epidemiologic observations. Sequencing performance differed across species, with HCoV-OC43 and HCoV-NL63 generally achieving broader genome recovery and more consistent sequencing output than HCoV-229E and especially HCoV-HKU1. Although Ct value is often expected to influence genome recovery, the correlation between Ct and genome breadth or mean depth was not statistically significant in this dataset. This indicates that sequencing success depended not only on viral load but also on virus-specific recovery characteristics. Potential contributors include species-specific probe performance, RNA integrity, genome composition, sample handling, stochastic library effects, and uneven read distribution across the genome. These differences were reinforced by the genome-wide depth profiles observed in the study, where HCoV-OC43 and HCoV-NL63 showed higher and more even read coverage across large portions of the genome, whereas HCoV-229E and HCoV-HKU1 displayed lower and more heterogeneous coverage. The recurrent increase in coverage toward the 3′ end observed in several profiles is also consistent with known compositional and sequencing-related biases in RNA viruses and underscores the importance of examining depth patterns before downstream interpretation [51].

The phylogenetic analyses further revealed clear differences in lineage diversity across endemic HCoV species. All recovered HCoV-229E genomes clustered within genotype L6, and all recovered HCoV-HKU1 genomes grouped within genotype A, suggesting relatively limited diversity among the genomes obtained for these two species. In contrast, HCoV-NL63 genomes were distributed across genotypes B and C2, while HCoV-OC43 genomes clustered within genotypes F, I, and J, consistent with the co-circulation of multiple genetically distinct lineages. These contrasting patterns indicate heterogeneous evolutionary dynamics among endemic HCoVs in Côte d’Ivoire and illustrate the added value of whole-genome sequencing over partial-gene approaches for resolving lineage diversity in respiratory virus surveillance. However, because coronaviruses can undergo recombination and formal recombination screening was not performed in this study, these genotype assignments should be interpreted cautiously as reference-supported phylogenetic placements rather than definitive whole-genome evolutionary classifications. This caution is particularly important for species represented by small numbers of recovered genomes, especially HCoV-HKU1. Together with the increasing availability of coronavirus functional tools, including recombinant HCoV-OC43 systems, such genomic baselines may support future phenotypic follow-up of epidemiologically relevant lineages [52]. Although the present study focuses on endemic HCoVs rather than SARS-CoV-2, recent pediatric SARS-CoV-2 seroepidemiologic studies provide a useful comparator for interpreting coronavirus surveillance in children. In a pre-vaccination pediatric serosurveillance study including 3099 children, Filippatos et al. showed increasing SARS-CoV-2 seropositivity across successive pandemic waves, supporting the value of repeated seroepidemiologic monitoring for capturing cumulative coronavirus exposure in pediatric populations [53]. A subsequent study conducted during Delta and Omicron predominance further showed that pediatric SARS-CoV-2 seropositivity increased from 29.7% during the Delta period to 48.5% during the Omicron period [54]. Together, these data illustrate that coronavirus exposure patterns can change markedly across epidemic and variant periods and that molecular surveillance alone may underestimate cumulative infection burden when asymptomatic or untested infections are frequent. In the context of endemic HCoVs, these observations support complementary surveillance strategies combining molecular detection, genomic characterization, and, where feasible, repeated seroepidemiologic monitoring to better define age-specific exposure, infection burden, and temporal shifts in circulation.

Several limitations should be considered when interpreting these findings. Sequencing was performed on a selected subset of RT-qPCR-positive samples with relatively low Ct values, which increased the likelihood of successful genome recovery but limited the representativeness of the sequenced dataset. In addition, the number of recovered genomes differed across species, which may have influenced the apparent diversity observed in the phylogenetic analyses. Monthly and site-specific denominators were incomplete, limiting formal inference on seasonality, geographic variation, and changes in circulation intensity. Genome recovery metrics were available for only a subset of sequenced samples, and some species, especially HCoV-HKU1, were represented by small numbers of recovered genomes. In addition, interpretation of the sequencing results is limited by the need for transparent reporting of sequencing denominators, including the number of positive samples selected, libraries prepared, genomes recovered, and genomes retained for phylogenetic analysis. Accession numbers, phylogenetic support values, and expanded sample metadata are therefore important for improving reproducibility and comparability with future studies from the region.

## 5. Conclusions

In conclusion, this study establishes an important first genomic baseline for endemic HCoV in Côte d’Ivoire. By combining temporal surveillance data with genome recovery assessment and phylogenetic analysis, it shows that endemic HCoVs circulated year-round with species-specific temporal patterns, differed in sequencing performance, and exhibited contrasting levels of lineage diversity. These findings support the further evaluation and targeted use of genomic approaches, including hybrid-capture sequencing and within-routine respiratory virus surveillance, and improve the understanding of endemic HCoV circulation in Côte d’Ivoire.

## Figures and Tables

**Table 1:** Distribution of HCoV-positive cases by age group, 2022–2024.

Age Group	Tested N (%)	HCoV Positive *n* (%)	Positivity Rate (%) ^a^	95% CI
<1 year	3002 (22.2)	99 (22.6)	3.30	2.72–4.00
1–5 years	4620 (34.1)	155 (35.3)	3.35	2.87–3.91
6–15 years	1053 (7.8)	25 (5.7)	2.37	1.61–3.48
16–35 years	2238 (16.5)	80 (18.2)	3.57	2.88–4.43
36–65 years	2108 (15.6)	67 (15.3)	3.18	2.51–4.02
>65 years	509 (3.8)	13 (3.0)	2.55	1.50–4.32
Total	13,530	439	3.24	2.96–3.56

**^a^** Positivity rates were calculated as the number of HCoV-positive samples divided by the number tested in each age group. Confidence intervals were estimated using binomial methods. Because this analysis was descriptive and not adjusted for year, month/season, site/region, sex, or clinical syndrome, age-group comparisons should not be interpreted as independent risk associations. Odds ratios and reference-category-based comparisons were not emphasized because the >65-year age group included only 13 positive cases.

**Table 2:** Species-by-year distribution of HCoV-positive cases selected for sequencing, 2022–2024.

Species	2022	2023	2024	Total Selected
HCoV-NL63	9	3	2	14
HCoV-229E	6	0	4	10
HCoV-OC43	8	3	7	18
HCoV-HKU1	1	5	4	10
Total	24	11	17	52

## Data Availability

The data generated and analyzed during this study derive from the national respiratory virus surveillance system of Côte d’Ivoire and are subject to institutional and public health data-governance restrictions. Until the generated sequences are deposited in a public data repository, de-identified surveillance data, consensus sequences, and associated metadata supporting the findings of this study will be made available by Dr Herve A. Kadjo upon reasonable request and subject to approval by the Institut Pasteur de Côte d’Ivoire and the relevant national public health authorities.

## Supplementary Materials

The following supporting information can be downloaded at: https://www.mdpi.com/article/10.3390/v18060678/s1, Supplementary Table S1: Reference and study sequences used for phylogenetic reconstruction of endemic HCoVs. The table lists all Côte d’Ivoire study sequences and publicly available reference sequences included in the maximum-likelihood phylogenetic analyses for HCoV-229E, HCoV-HKU1, HCoV-NL63, and HCoV-OC43. For each sequence, the table provides the virus species, sequence type, accession or sample identifier, corrected tree label, country or location, collection year, genotype or clade placement in the corrected tree, sequence length, inclusion status, rationale for inclusion, and notes where applicable. Reference sequences were selected to represent recognized genotype diversity, geographic and temporal diversity, and publicly available genomes clustering near the Côte d’Ivoire sequences. For Côte d’Ivoire sequences, genotype placement was inferred from clustering with genotype-labeled reference sequences in the corrected phylogenetic trees; Supplementary Table S2: Metadata and sample-level sequencing metrics for the 52 endemic HCoV-positive samples selected for targeted hybrid-capture sequencing. The table includes sample identifier, year, HCoV species, Ct value, clinical syndrome, health facility, sequencing status, and, where available, total reads, HCoV-mapped reads, mapped-read proportion, genome breadth, mean depth, and recovery category. Mapped-read proportion was calculated as HCoV-mapped reads divided by total reads × 100.
